# Why PRP works only on certain patients with tennis elbow? Is *PDGFB* gene a key for PRP therapy effectiveness? A prospective cohort study

**DOI:** 10.1186/s12891-021-04593-y

**Published:** 2021-08-18

**Authors:** Paweł Niemiec, Karol Szyluk, Anna Balcerzyk, Marcin Kalita, Alicja Jarosz, Joanna Iwanicka, Tomasz Iwanicki, Tomasz Nowak, Marius Negru, Tomasz Francuz, Wojciech Garczorz, Władysław Grzeszczak, Sylwia Górczyńska-Kosiorz, Wojciech Kania, Iwona Żak

**Affiliations:** 1 Department of Biochemistry and Medical Genetics, School of Health Sciences in Katowice, Medical University of Silesia in Katowice, Medyków 18 str, 40-752 Katowice, Poland; 2 District Hospital of Orthopaedics and Trauma Surgery, Bytomska 62 str, 41-940 Piekary Śląskie, Poland; 3 Trauma and Orthopaedics Departament, St. Bernard’s Hospital, Harbour Views Rd, GX11 1AA Gibraltar, UK; 4 Department of Biochemistry, School of Medicine in Katowice, Medical University of Silesia in Katowice, Medyków 18 str, 40-752 Katowice, Poland; 5 Department of Internal Medicine, Diabetology and Nephrology, School of Medicine, Division of Dentistry in Zabrze, Medical University of Silesia in Katowice, 41-800 Zabrze, Poland; 6 Department of Trauma and Orthopedic Surgery, Multidisciplinary Hospital in Jaworzno, Chełmońskiego 28 Str, 43-600, Jaworzno, Poland

**Keywords:** Platelet-rich plasma, Tennis elbow, Tendinopathy, *PDGFB*, single nucleotide polymorphisms, gene

## Abstract

**Background:**

There is variability in individual response to platelet-rich plasma (PRP) therapy in tennis elbow treatment. Genetic variation, especially within genes encoding growth factors may influence the observed inter-individual differences. The purpose of this study was to identify polymorphic variants of the platelet-derived growth factor beta polypeptide gene (*PDGFB*) that determine an improved individual response to PRP therapy in tennis elbow patients.

**Methods:**

This prospective cohort study was designed in accordance with STROBE and MIBO guidelines. A cohort of 107 patients (132 elbows, 25 bilateral) was studied, including 65 females (77 elbows) and 42 males (55 elbows), aged 24–64 years (median 46.00 ± 5.50), with lateral elbow tendinopathy treated with autologous PRP injection. The effectiveness of PRP therapy was recorded in all subjects at 2, 4, 8, 12, 24 and 52 weeks after PRP injection using the Visual Analog Scale (VAS), quick version of Disabilities of the Arm, Shoulder and Hand score (QDASH) and Patient-Rated Tennis Elbow Evaluation (PRTEE). In order to determine the *PDGFB* variants with the best response to PRP therapy, patient reported outcome measures were compared between individual genotypes within studied polymorphic variants (rs2285099, rs2285097, rs2247128, rs5757572, rs1800817 and rs7289325). The influence of single nucleotide polymorphisms on blood and PRP parameters, including the concentration of PDGF-AB and PDGF-BB proteins was also analyzed.

**Results:**

Our analysis identified genetic variants of the *PDGFB* gene that lead to a better response to PRP therapy. The TT (rs2285099) and CC (rs2285097) homozygotes had higher concentration of platelets in whole blood than carriers of other genotypes (*p* = 0.018) and showed significantly (*p* < 0.05) lower values of VAS (weeks 2–12), QDASH and PRTEE (weeks 2–24). The rs2285099 and rs2285097 variants formed strong haplotype block (r^2^ = 98, D’=100). The AA homozygotes (rs2247128) had significantly lower values of VAS (weeks 4–52), QDASH and PRTEE (weeks 8, 12).

**Conclusions:**

*PDGFB* gene’s polymorphisms increase the effectiveness of PRP therapy in tennis elbow treatment. Genotyping two polymorphisms of the *PDGFB* gene, namely rs2285099 (or rs2285097) and rs2247128 may be a helpful diagnostic tool while assessing patients for PRP therapy and modifying the therapy to improve its effectiveness.

**Supplementary Information:**

The online version contains supplementary material available at 10.1186/s12891-021-04593-y.

## Background

Platelet-rich plasma (PRP) has been used as a minimally invasive treatment for lateral elbow tendinopathy for many years. Multiple studies showed its effectiveness. [1–3] However, there is variability in the individual response to PRP therapy. Although this method can improve pain and functional outcome, there is no therapeutic effect in some cases. In addition to demographic factors modulating the effectiveness of PRP therapy in the treatment of tendinopathies,[4–6] genetic factors may influence the observed inter-individual differences. Single nucleotide variants (SNVs), including single nucleotide polymorphisms (SNPs) and point mutations, account for 90 % of the variability in the human genome.[7] Due to biological role of growth factors, their genes as well as the genes encoding their receptors seem to be the best markers of the regenerative process.[8] These genes are highly polymorphic [9] and may affect inter-individual variability of the regeneration rate. Platelet-derived growth factor (PDGF) is one of the key growth factors, integrating processes leading to the regeneration of bones and soft tissues.[10–12] Due to its pleiotropic properties, PDGF is directly involved in angiogenesis processes, also activating mesenchymal differentiation and osteogenesis.[10, 11, 13, 14] Platelets produce and release PDGF-AB heterodimer, PDGF-AA and PDGF-BB homodimers molecules.[15] Subunits of platelet isoforms of PDGF proteins are encoded by genes *PDGFA* (7p22.3)[16] and *PDGFB* (22q13.1),[17] respectively.

The biological role of PDGF proteins raises the question of whether their genes may have an impact on the effectiveness of the treatment of musculoskeletal system injuries with the use of PRP. Therefore, we designed and conducted the present study mainly to search for the *PDGFB* gene variants that can improve individual response to PRP therapy in patients with tennis elbow.

## Methods

### Study design

This prospective cohort study was designed in accordance with STROBE and MIBO guidelines. The patients were followed up for one year and common patient-reported outcome measures (PROMs) were recorded. Six single nucleotide polymorphisms of the *PDGFB* gene were genotyped and the effectiveness of PRP therapy was compared between genotypic variants. The influence of SNPs on blood and PRP parameters, including the concentration of PDGF-AB and PDGF-BB proteins was also analyzed.

### Patients

A cohort of 107 patients (132 elbows, 25 bilateral, 100 %) was studied, including 65 females (77 elbows, 58.3 %) and 42 males (55 elbows, 41.7 %), aged 24–64 years (median 46.00 ± 5.50), with lateral elbow tendinopathy treated with autologous PRP injection.

**Fig. 1:**
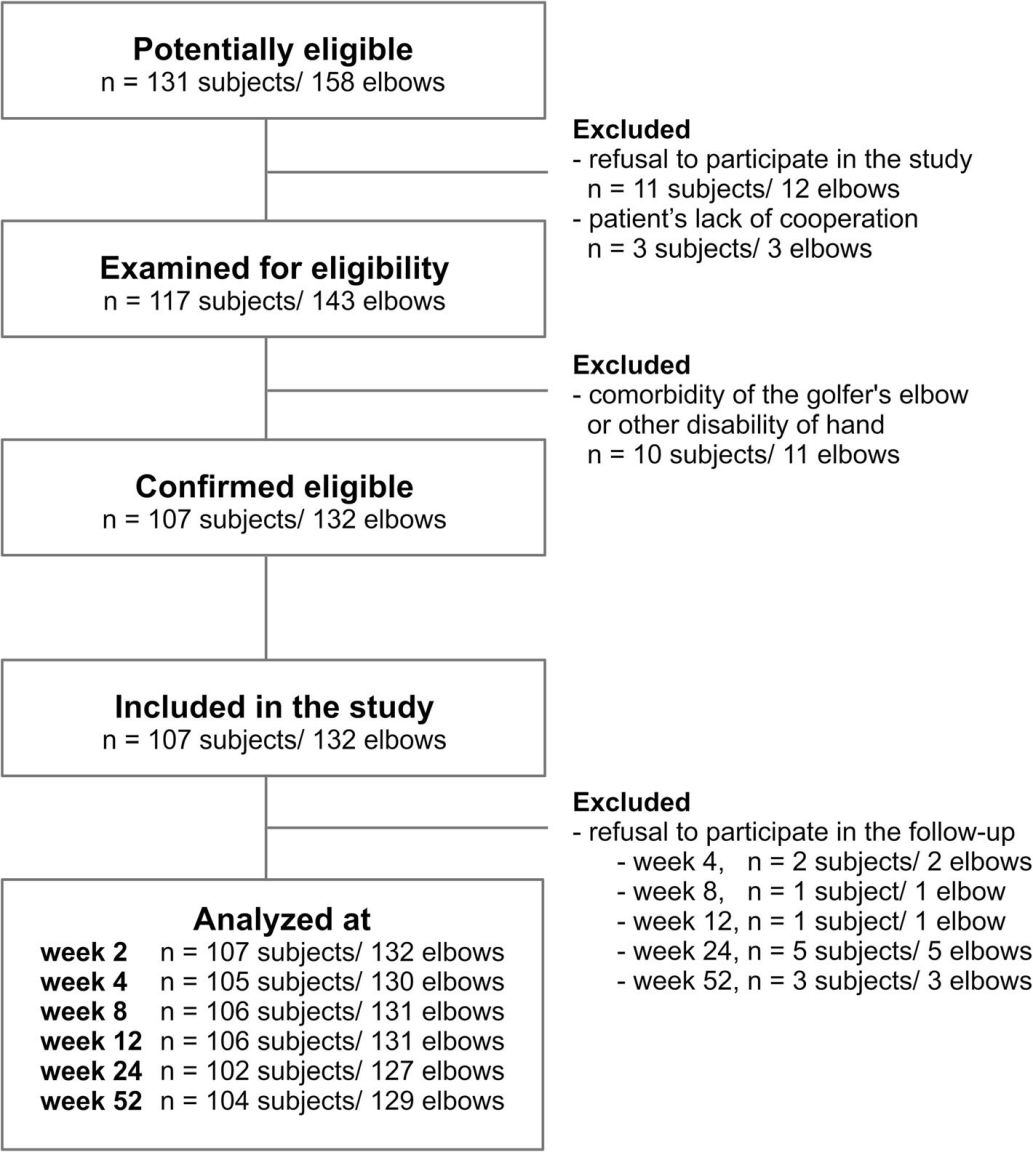
Flowchart of the study selection

Patients were enrolled to the study between November 2018 and November 2019, and they were selected for the study, examined and injected by the same orthopaedic surgeons, following the study protocol. Follow up data was collected until November 2020. Flow diagram of the patients included in the study is presented below (Fig. 1).

The inclusion criteria was the clinical diagnosis of lateral epicondylitis: pain in the region of common extensor origin radiating distally and proximally, weakening the grip strength, pain and muscle weakness increasing when holding and lifting items, morning stiffness, repeated activities and/ or limb overuse, positive Thomson’s, Mill’s tests and Cozen’s sign, tenderness at palpation over the lateral epicondyle of the humerus. At least three months duration of symptoms before injection. The exclusion criteria were: additional injury/ disease of affected limb, prior surgical intervention, rheumatoid arthritis, pregnancy, active malignancy, cervical radiculopathy, current anti-platelet medication, local steroid injections in the preceding 6 months, previous PRP injections, cognitive limitations. There was no formal post-injection rehabilitation protocol. Further post injection therapy (physiotherapy, nonsteroidal anti-inflammatory drugs, steroids, additional injections of PRP) was monitored during the follow-up period but was not considered a criteria for exclusion.

### PRP separation and injection procedure

Blood collection, separation and injection of PRP were performed in standardized conditions (20^o^C, same light exposure). PRP was separated from fresh whole blood, immediately after blood collection as per manufacturer’s instructions (Autologous Conditioned Plasma, Arthrex GmbH, Germany). A 1.2 mm needle was used to collect 12 ml of whole blood. Blood was mixed with 3.13 % sodium citrate (MediPac GmbH, Germany) in the proportion of 9:1 and then centrifuged under the same conditions using a Rotofix 32 A centrifuge (Andreas Hettich GmbH & Co, Germany). The centrifugation process was carried out at a speed of 1500 rpm and the spinning time was 5 min. Between 2.5 and 3.5 ml of PRP was collected and a volume of 2.0 to 3.0 ml was injected immediately after centrifugation in the common extensor origin area using a 1.2 mm needle. The injection was performed under ultrasound guidance using the Mindray DC-3 apparatus with a linear probe with a frequency range of 5, 7.5, 10 MHz. The remaining 0.5 ml of PRP was saved for further analysis. All procedures were performed in an outpatient clinic in the treatment room with staff nurse in the room, using disposable equipment. The injections were performed directly by two Senior Trauma and Orthopedic Consultants (KS and WK) with 17 years of experience.

After the procedure, each patient was observed for 30 min in the clinic, attention was paid to any possible complications. In the absence of disturbing local and general symptoms, the patient was discharged. Each patient was advised to contact the hospital in case of any adverse reactions. Patients were also advised to avoid heavy use of the affected limb for 24 h. No infection at the injection site was observed in any of the patients.

### Whole blood and PRP parameters

On the day of PRP injection, full blood count and the measures of platelets, plateletcrit (PCT), mean platelet volume (MPV) and platelet distribution width (PDW) in fresh PRP were determined. The remaining 0.5 ml of PRP was aliquoted to small tubes to avoid redundant freeze-thaw cycles, then frozen at -86oC. The Human DuoSet ELISA kits for PDGF-AB and PDGF-BB (R&D Systems Inc., MN, USA) were used to assess the concentrations of the proteins. The ELISA tests were performed in triplicate for each PRP sample. For patients with bilateral tennis elbow, if the injections were carried out on different dates, separate analyzes (whole blood, PRP) were performed.

### Follow-up, outcomes, measures of effectiveness

The effectiveness of PRP therapy was analyzed and compared to clinical condition (pain and functionality/ disability of affected limb) on the day of injection (baseline, week 0). Follow up review was performed at 2, 4, 8, 12, 24 and 52 weeks. Follow-up will be continued for two years after injection.

VAS, QDASH and PRTEE questionnaires were used for assessment of pain and disability, with the following ranges assumed: 0 minimum and 10 maximum pain for VAS, 0 minimum and 100 maximum pain/ disability for QDASH and PRTEE. The effectiveness of therapy in relation to individual variables was determined based on raw values of outcomes (VAS, QDASH, PRTEE) and the differences of outcomes vs. baseline (ΔVAS, ΔQDASH and ΔPRTEE). If the differences of raw PROMs were statistically significant at baseline, the latter measures were considered.

### Genetic analyses

Genomic DNA was isolated from peripheral blood leukocytes using the MasterPure genomic DNA purification kit (Epicenter Technologies, WI, USA). SNPs of the *PDGFB* gene were genotyped using the TaqMan Predesigned SNP Genotyping Assay kits and the 7300 Real-Time PCR System (Thermo Fisher Scientific, CA, USA). The accuracy of genotyping was checked by re-genotyping 10–15 % of samples. Repeatability of results reached 100 %. Genetic analyzes were performed by qualified molecular biologists with at least 15 years of laboratory experience.

Only SNPs with minor allele frequency ≥ 20 % in populations of European origin, based on the Database of SNPs of National Center for Biotechnology Information, U.S. National Library of Medicine,[9] were selected for analysis. There were rs2285099 (C > T), rs2285097 (T > C), rs2247128 (A > G), rs5757572 (C > G), rs1800817 (G > T) and rs7289325 (A > T) variants. The first five are intronic polymorphisms, the sixth is located in the 5’ untranslated region (5’ UTR) of the *PDGFB* gene.

### Statistical analyses

Data were analyzed using the Statistica 13.0 software (TIBCO Software Inc, CA, USA). Normality of distribution of quantitative data was assessed by the Shapiro-Wilk test and comparisons were performed using the Mann-Whitney U test (all variables had non-normal distribution). All quantitative data were given as a median, and their spread as a quartile deviation (QD). Pearson’s r coefficient was used to interpret the strength of correlation between quantitative variables. Cases with missing data were rejected from the respective comparisons.

Genetic data were analyzed in dominant, recessive and additive models of inheritance. The Hardy-Weinberg equilibrium was tested by a χ2 test as well as comparisons of genotypes and alleles frequencies between groups differentiated by qualitative variables. Fisher’s correction was used for subgroups with less than ten subjects. Haplotype blocks were defined by the HaploView software [18] using the Gabriel et al. algorithm.[19] D’ and r2 values were used as linkage disequilibrium measures. Study size and power analysis were computed using the Epi-InfoTM 7.2.1.0 software (Centers for Disease Control and Prevention, USA). Statistical significance was accepted at *p* < 0.05. In cases of multiple comparisons, the p values were adjusted using the Bonferroni correction.

## Results

### General characteristics of the study group

Demographic and clinical characteristics of the group are presented in the Table 1.

**Table 1:** Demographic and clinical characteristics of the study group at baseline (week 0)

Characteristics			
General	number of subjects, N	107	-
	number of elbows, n (%)	132	(100.0)
	tennis elbow in dominant hand, n (%)	86	(65.2)
	tennis elbow in non-dominant hand, n (%)	46	(34.8)
	females, n (%)	77	(58.3)
	age, median ± QD	46.00	5.50
	BMI, median ± QD	25.65	2.00
	overweight/obesity BMI ≥ 25, n (%)	86	(65.2)
	current smokers, n (%)	22	(16.6)
	former smokers, n (%)	48	(36.4)
Comorbidities	diabetes mellitus, n (%)	4	(3.0)
	gout, n (%)	8	(6.1)
	thyroid diseases, n (%)	15	(11.4)
	hypercholesterolaemia, n (%)	7	(5.3)
	hypertension, n (%)	18	(13.6)
	heart failure, n (%)	4	(3.0)

Demographic factors, BMI, stimulants or comorbidities did not affect the values of PROMs (*p* > 0.05)

### Platelets and PDGF proteins concentration

The median platelet concentration (10^9^/l ± QD) in the whole blood was 240.00 ± 40.50 (Table 2), being higher in females than in males (261.50 ± 33.00 vs. 224.00 ± 38.75, respectively, *p* < 0.001). The concentration of platelets in PRP was similar in men and women. PDGF-AB and PDGF-BB concentrations were higher among patients with BMI ≥ 25 and cigarette smokers (only PDGF-AB). PDGF-AB and PDGF-BB concentrations correlated with the concentration of platelets in PRP (*r* = 0.72, *p* < 10^− 6^ and *r* = 0.42, *p* < 0.001, respectively) and with each other (*r* = 0.72, *p* < 10^− 6^). There was also a weak correlation between the concentration of PDGF-AB and the concentration of platelets in whole blood (*r* = 0.25, *p* = 0.01). PDGF-AB, PDGF-BB and platelets concentrations didn’t correlate with PROMs values.

**Table 2:** Platelets parameters in whole blood and PRP preparation of patients

Parameter	Whole blood		PRP	
	median	± QD	median	± QD
PLT 10^9^/l	240.00	40.50	343.00	65.00
PCT ml/l	2.31	0.36	0.30	0.06
MPV fl.	9.10	0.73	8.60	0.40
PDW fl.	16.10	0.15	14.60	0.25
WBC 10^9^/l	6.26	1.16	-	-
RBC 10^12^/l	4.67	0.30	-	-
PDGF-AB ng/ml	-	-	8.27	2.40
PDGF-BB ng/ml	-	-	4.64	1.45

### Additional treatment

Over 52 weeks of follow-up, the following additional treatment was implemented: steroid injection (n = 7 patients/ 9 elbows, 6.8 %), non-steroidal anti-inflammatory drugs (n = 28 patients/ 33 elbows, 25.0 %), additional PRP injection (n = 10 patients/ 10 elbows, 7.6 %), physical therapy (n = 28 patients/ 35 elbows, 26.5 %), manual therapy (n = 22 patients/ 27 elbows, 20.5 %), kinesitherapy (n = 3 patients/ 3 elbows, 2.3 %) and arthroscopy (n = 3 patients/ 4 elbows, 3.0 %). Additional forms of therapy didn’t affect the values of PROMs, except of physical therapy. Patients treated with physical therapy methods showed worse therapeutic efficacy (significantly higher values of VAS, QDASH and PRTEE) at weeks 8–24, than untreated patients. higher values of VAS, QDASH and PRTEE, *p* < 0.05) at weeks 8–24, than untreated patients.

### Analysis of the ***PDGFB*** gene polymorphisms

Genotyping data were obtained for 107 subjects (132 elbows) (Table 3). The exception is the rs1800817 polymorphism, in which genotyping was unsuccessful in two patients. Genotype frequencies of all SNPs were compatible with the Hardy-Weinberg equilibrium (*p* > 0.05).

**Table 3:** Frequency of genotypes and alleles of analyzed SNPs of the *PDGFB* gene

SNP	Position onchromosome 22	Genotypes	n (%) ^a^	Alleles	n (%) ^a^
rs2285099	39,226,053	CC	51 (38.6)	C	163 (61.7)
		CT	61 (46.2)	T	101 (38.3)
		TT	20 (15.2)		
rs2285097	39,226,329	TT	50 (37.9)	T	162 (61.4)
		CT	62 (47.0)	C	102 (38.6)
		CC	20 (15.1)		
rs2247128	39,234,282	GG	73 (55.3)	G	191 (72.3)
		AG	45 (34.1)	A	73 (27.7)
		AA	14 (10.6)		
rs5757572	39,236,915	GG	46 (34.8)	G	161 (61.0)
		CG	69 (52.3)	C	103 (39.0)
		CC	17 (12.9)		
rs1800817	39,243,848	TT	72 (55.4)	T	192 (73.8)
		GT	48 (36.9)	G	68 (26.2)
		GG	10 (7.7)		
rs7289325	39,246,572	TT	24 (18.2)	T	117 (44.3)
		AT	69 (52.3)	A	147 (55.7)
		AA	39 (29.5)		

^a^ Percentage of analyzed elbows (*N* = 132, except of rs1800817 where *N* = 130)

**Fig. 2:**
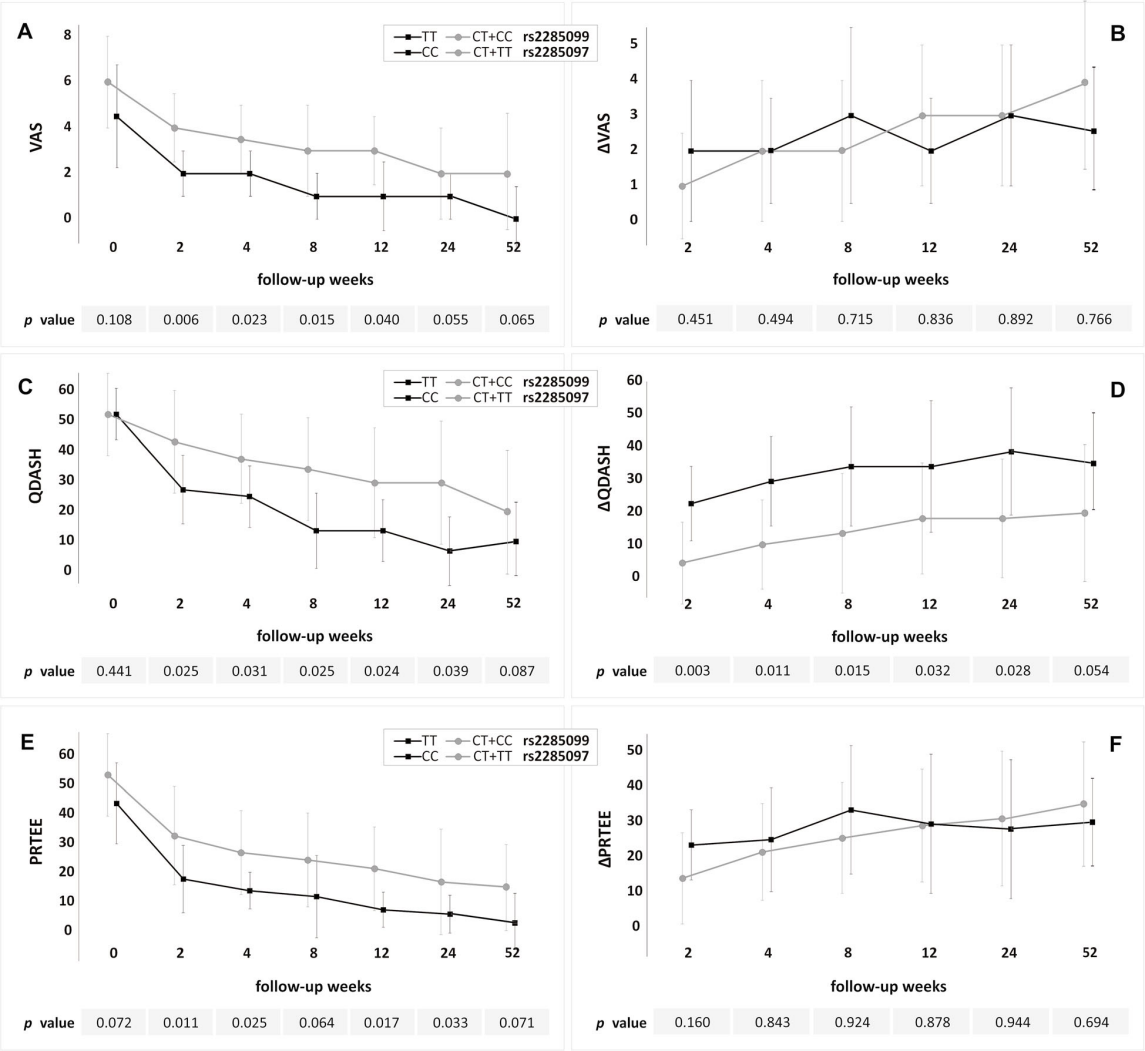
Medians (± QD) of patient reported outcome measures: VAS (**A**), ΔVAS (**B**), QDASH (**C**), ΔQDASH (**D**), PRTEE (**E**) and ΔPRTEE (**F**) in respect to rs2285099 and rs2285097 SNPs variants

**Fig. 3:**
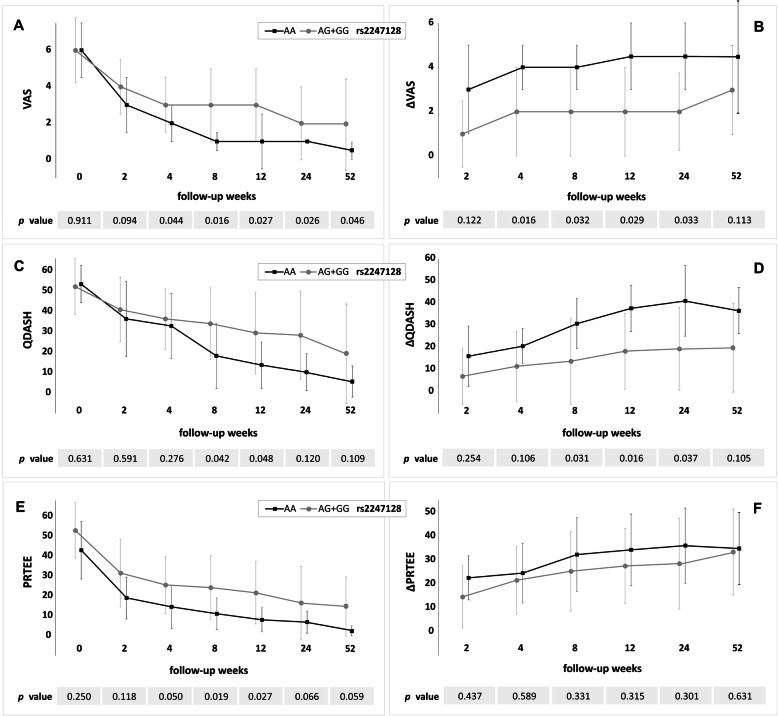
Medians (± QD) of patient reported outcome measures: VAS (**A**), ΔVAS (**B**), QDASH (**C**), ΔQDASH (**D**), PRTEE (**E**) and ΔPRTEE (**F**) in respect to rs2247128 SNP variants

The rs2285099 and rs2285097 SNPs variants form strong haplotype block (r^2^ = 98, D’=100), so they will be discussed together. The TT (rs2285099) and CC (rs2285097) homozygotes showed significantly lower values of VAS (weeks 2–12), QDASH (weeks 2–24) and PRTEE (weeks 2, 4, 12 and 24) as well as higher ΔQDASH (weeks 2–24) (Fig. 2, additional files 1 and 2). These homozygotes also had significantly higher concentration of platelets in whole blood than the carriers of the other genotypes (*p* = 0.018) (additional files 1 and 2). The AA homozygotes of the rs2247128 had significantly lower values of VAS (weeks 4–52), QDASH (weeks 8, 12) and PRTEE (weeks 8, 12) as well as higher values of ΔVAS (weeks 4–24) and ΔQDASH (weeks 8–24) than G allele carriers (Fig. 3, additional file 3).

The CC homozygotes of the rs5757572 had significantly lower values of VAS (week 24), PRTEE (weeks 24, 52) and higher values of ΔVAS (week 24) and ΔQDASH (week 24) than G allele carriers (additional file 4). The GG homozygotes of the rs1800817 had significantly lower values of all scores (VAS, QDASH and PRTEE) at week 24 and higher values of ΔVAS and ΔQDASH also at week 24 (additional file 5). Variants of the rs7289325 polymorphism did not affect the values of PROMs (additional file 6).

There were no statistically significant differences in the distribution or median of variables like age, sex, cigarette smoking, BMI, PDGF proteins concentrations, additional treatment (including physical therapy) and comorbidities between genotypic groups of respective SNPs (*p* > 0.025, after Bonferroni correction)

## Discussion

In the present study we identified polymorphic variants of the *PDGFB* gene which increase the effectiveness of PRP therapy in tennis elbow treatment. Better outcome of PRP therapy regarded TT (rs2285099)/ CC (rs2285097) and AA (rs2247128) homozygotes (weeks 2–24 and weeks 4–52 of follow-up, respectively). SNVs of rs5757572 and rs1800817 were also associated with an improvement in PROMs during multiple follow ups (week 24 and 52). SNPs were the only factors related to the effectiveness of the therapy in the present study (sex, age, BMI, cigarette smoking or comorbidities did not affect the values of pain/ disability). Based on statistical analyses results the s2285099/ rs2285097 and rs2247128 polymorphism variants improved the most the efficacy of PRP. Statistical significance of differences between the genotypes of the remaining *PDGFB* gene polymorphisms decreased with their distance from rs2285099, rs2285097 and rs2247128 and the proximity of the 5 ‘UTR of the *PDGFB* gene. The variants of the rs7289325 polymorphism (as the only one located outside the *PDGFB* gene at the greatest distance on chromosome 22 from the rs2285099, rs2285097 and rs2247128 SNPs), did not influence the efficacy of PRP therapy.

Based on the current knowledge, PDGF is a strong mitogenic factor for cells involved in the repair of musculoskeletal tissue, namely mesenchymal stem cells, osteogenic cells and tenocytes.[10–12, 14, 20, 21] For the above reasons, it is surprising that the influence of the inter-individual variability of the *PDGFB* gene on the effectiveness of the treatment of injuries of the musculoskeletal system has not been studied so far. Moreover, the current state of research does not even explain the influence of the polymorphisms analyzed in this study on the functioning of the *PDGFB* gene and PDGF proteins. Probably also such research has not yet been undertaken. Literature data regarding SNPs are fragmentary and usually come from case-control studies on different phenotypes, mainly cancers. For example, the variants of rs5757572, rs1800817 and rs2247128 SNPs which improved the results of PROMs were previously associated with increased risk of pancreatic cancer,[22] gall bladder cancer [23] and breast cancer.[24] The fact that these variants are risk factors of the neoplastic process, may suggest that they are rather markers of PDGF-dependent proliferative potential of cells, which would partly explain the observed results. The influence of the analyzed SNPs on the functionality of the *PDGFB* gene requires further research, as well as determining the relationship between their variants and the concentrations of PDGF proteins and other platelet parameters. Even though we observed higher levels of platelets (in whole blood) and higher concentrations of PDGF-AB protein (in PRP) in homozygotes with higher therapeutic effectiveness, differences in platelet concentration were statistically significant between genotypes of only two analyzed SNPs, namely rs2285099 and rs2285097.

Available studies investigating the relationship between genetic factors and injuries of the musculoskeletal system [25–27] are usually case-control studies focused on the search for risk factors for trauma and did not analyze their influence on the effectiveness of therapy over time. Such studies are still missing, and only their results provide knowledge about genetic factors that potentially improve the treatment of injuries of the musculoskeletal system. For those reasons current research provides a new look at the role of genetic testing in the therapeutic process.

The weaknesses of the current research are: relatively small size of the studied group, short follow up (one year) and lack of formal post-injection rehabilitation protocol. The group size, too low in the case of qualitative traits studies (e.g. association case-control studies) was, however, sufficient for the analysis of quantitative outcomes like PROMs. As for the observation period, our target is two years of follow-up. Although lack of a unified rehabilitation protocol may contribute to a potential disturbance in the observed outcomes, we found that the therapies did not improve PROMs values and the frequencies of *PDGFB* genotypes did not differentiate between patients treated and untreated with respective types of therapies. We want to emphasize that our decision to not apply the formal rehabilitation protocol was dictated by professional ethics according to which we could not prohibit patients from accessing other forms of therapy.

Many orthopaedic surgeons are looking for a reliable way to predict which patients with tendinopathies or other injuries will benefit from the PRP injection treatment and the results of our research indicate the presence of potential diagnostic tool for identify these patients and increase the success rate of this largely used treatment. This may be of great interest for the community of physicians, physiotherapists, athletic trainers and anyone involved in treating tennis elbow and other injuries of musculoskeletal system. At the current stage of research, it seems that genotyping two polymorphisms of the *PDGFB* gene, namely rs2285099 (or rs2285097) and rs2247128 would be a helpful diagnostic tool to typing patients for PRP therapy and modifying the therapy to improve its effectiveness in Caucasian patients. This does not change the fact that determining their influence on efficacy and on the role in the regeneration process requires further functional and clinical studies.

In conclusion, the effectiveness of PRP treatment of tennis elbow depends on the *PDGFB* gene polymorphism. Variants of the *PDGFB* gene SNPs, namely rs2285099/ rs2285097 and rs2247128 improve the response to PRP therapy in patients with tennis elbow treated with PRP.

## Conclusions

PPDGF is a strong mitogenic factor, but so far there is no evidence that *PDGF* genes variability influence the efficacy of treating musculoskeletal system injuries with PRP .

We found that the effectiveness of PRP treatment of tennis elbow depends on the *PDGFB* gene polymorphism.

Variants of the *PDGFB* gene SNPs, namely rs2285099/ rs2285097 and rs2247128 improve the response to PRP therapy in patients with tennis elbow treated with PRP.

An identification of genetic factors that influence the effectiveness of treatment with PRP may find clinical application in the future, e.g. when assessing patients for PRP therapy and modifying the therapy to improve its outcome.

Genotyping two polymorphisms of the *PDGFB* gene, namely rs2285099 (or highly linked rs2285097) and rs2247128 would be a helpful diagnostic tool to achieve the above goals.

## Supplementary Information

**Additional file 1:** Platelets parameters and pain scores values in TT homozygotes and C allele carriers of the rs2285099 *PDGFB* gene polymorphism.

**Additional file 2:** Platelets parameters and pain scores values in CC homozygotes and T allele carriers of the rs2285097 *PDGFB* gene polymorphism.

**Additional file 3:** Platelets parameters, pain scores values and other characteristics differentiating AA homozygotes and G allele carriers of rs2247128 *PDGFB* gene polymorphism.

**Additional file 4:** Platelets parameters and pain scores in CC homozygotes and G allele carriers of the rs5757572 *PDGFB* gene polymorphism.

**Additional file 5:** Platelets parameters and pain scores values in GG homozygotes and T allele carriers of the rs1800817 *PDGFB* gene polymorphism.

**Additional file 6:** Platelets parameters and pain scores values in AA homozygotes and T allele carriers of the rs7289325 *PDGFB* gene polymorphism.

## Data Availability

All data relevant to the study are included in the article or uploaded as online supplementary information. The access to anonymized raw data can be obtained from the corresponding author on reasonable request.

## Abbreviations

PRP: Platelet-rich plasma
PDGFB: Platelet-derived growth factor beta polypeptide
VAS: Visual Analog Scale
QDASH: Disabilities of the Arm, Shoulder and Hand score
PRTEE: Patient-Rated Tennis Elbow Evaluation
SNV: Single nucleotide variants
SNP: Single nucleotide polymorphisms
PDGF: Platelet-derived growth factor
PROM: Patient-reported outcome measure
PLT: Platelets
PCT: Plateletcrit
MPV: Mean platelet volume
PDW: Platelet distribution width
RBC: Red blood cells
WBC: White blood cells
QD: Quartile deviation
BMI: Body mass index

## References

[1] Dong W, Goost H, Lin X, Burger C, Paul C, Wang Z, Kong F, Welle K, Jiang Z, Kabir K (2016). Injection therapies for lateral epicondylalgia: a systematic review and Bayesian network meta-analysis. Br J Sports Med..

[2] Fitzpatrick J, Bulsara M, Zheng MH (2017). The Effectiveness of Platelet-Rich Plasma in the Treatment of Tendinopathy: A Meta-analysis of Randomized Controlled Clinical Trials. Am J Sports Med.

[3] Lenoir H, Mares O, Carlier Y (2019). Management of lateral epicondylitis. Orthop Traumatol Surg Res.

[4] Miller LE, Parrish WR, Roides B, Bhattacharyya S. Efficacy of platelet-rich plasma injections for symptomatic tendinopathy: systematic review and meta-analysis of randomised injection-controlled trials. BMJ Open Sport Exerc Med. 2017; e000237. 10.1136/bmjsem-2017-000237. 10.1136/bmjsem-2017-000237 PMC5687544 29177072

[5] Salini V, Vanni D, Pantalone A, Abate M (2015). Platelet Rich Plasma Therapy in Non-insertional Achilles Tendinopathy: The Efficacy is Reduced in 60-years Old People Compared to Young and Middle-Age Individuals. Front Aging Neurosci.

[6] Xiong G, Lingampalli N, Koltsov JCB, Leung LL, Bhutani N, Robinson WH, Chu CR (2018). Men and Women Differ in the Biochemical Composition of Platelet-Rich Plasma. Am J Sports Med.

[7] Visscher PM, Wray NR, Zhang Q, Sklar P, McCarthy MI, Brown MA, Yang J (2017). 10 Years of GWAS Discovery: Biology, Function, and Translation. Am J Hum Genet.

[8] Pruna R, Til L, Artells R (2014). Could single nucleotide polymorphisms influence on the efficacy of platelet-rich plasma in the treatment of sport injuries?. Muscles Ligaments Tendons J.

[9] National Library of Medicine (US): National Center for Biotechnology Information. https://www.ncbi.nlm.nih.gov/snp/. Accessed Feb 2017 and 10 Mar 2021.

[10] Andrae J, Gallini R, Betsholtz C (2008). Role of platelet-derived growth factors in physiology and medicine. Genes Dev.

[11] Shah P, Keppler L, Rutkowski J (2014). A review of platelet derived growth factor playing pivotal role in bone regeneration. J Oral Implantol.

[12] Deptuła M, Karpowicz P, Wardowska A, Sass P, Sosnowski P, Mieczkowska A, Filipowicz N, Dzierżyńska M, Sawicka J, Nowicka E, Langa P, Schumacher A, Cichorek M, Zieliński J, Kondej K, Kasprzykowski F, Czupryn A, Janus Ł, Mucha P, Skowron P, Piotrowski A, Sachadyn P, Rodziewicz-Motowidło S, Pikuła M (2020). Development of a Peptide Derived from Platelet-Derived Growth Factor (PDGF-BB) into a Potential Drug Candidate for the Treatment of Wounds. Adv Wound Care (New Rochelle).

[13] Mariani E, Pulsatelli L (2020). Platelet Concentrates in Musculoskeletal Medicine. Int J Mol Sci.

[14] Liang T, Zhu L, Gao W, Gong M, Ren J, Yao H, Wang K, Shi D (2017). Coculture of endothelial progenitor cells and mesenchymal stem cells enhanced their proliferation and angiogenesis through PDGF and Notch signaling. FEBS Open Bio.

[15] Hart CE, Bailey M, Curtis DA, Osborn S, Raines E, Ross R, Forstrom JW (1990). Purification of PDGF-AB and PDGF-BB from human platelet extracts and identification of all three PDGF dimers in human platelets. Biochemistry.

[16] Betsholtz C, Johnsson A, Heldin CH, Westermark B, Lind P, Urdea MS, Eddy R, Shows TB, Philpott K, Mellor AL, Knott TJ, Scott J (1986). cDNA sequence and chromosomal localization of human platelet-derived growth factor A-chain and its expression in tumour cell lines. Nature.

[17] Collins T, Ginsburg D, Boss JM, Orkin SH, Pober JS (1985). Cultured human endothelial cells express platelet-derived growth factor B chain: cDNA cloning and structural analysis. Nature.

[18] Barrett JC, Fry B, Maller J, Daly MJ (2005). Haploview: analysis and visualization of LD and haplotype maps. Bioinformatics.

[19] Gabriel SB, Schaffner SF, Nguyen H, Moore JM, Roy J, Blumenstiel B, Higgins J, DeFelice M, Lochner A, Faggart M, Liu-Cordero SN, Rotimi C, Adeyemo A, Cooper R, Ward R, Lander ES, Daly MJ, Altshuler D (2002). The structure of haplotype blocks in the human genome. Science.

[20] Casati L, Celotti F, Negri-Cesi P, Sacchi MC, Castano P, Colciago A (2014). Platelet derived growth factor (PDGF) contained in Platelet Rich Plasma (PRP) stimulates migration of osteoblasts by reorganizing actin cytoskeleton. Cell Adh Migr.

[21] Evrova O, Buschmann J (2017). In vitro and in vivo effects of PDGF-BB delivery strategies on tendon healing: a review. Eur Cell Mater.

[22] Yang W, Liu H, Duan B, Xu X, Carmody D, Luo S, Walsh KM, Abbruzzese JL, Zhang X, Chen X, Wei Q (2019). Three novel genetic variants in NRF2 signaling pathway genes are associated with pancreatic cancer risk. Cancer Sci.

[23] Mishra K, Behari A, Kapoor VK, Khan MS, Prakash S, Agrawal S (2015). Platelet Derived Growth Factor-B and Human Epidermal Growth Factor Receptor-2 Polymorphisms in Gall Bladder Cancer. Asian Pac J Cancer Prev.

[24] Slattery ML, John EM, Stern MC, Herrick J, Lundgreen A, Giuliano AR, Hines L, Baumgartner KB, Torres-Mejia G, Wolff RK (2013). Associations with growth factor genes (*FGF1*, *FGF2*, *PDGFB*, *FGFR2*, *NRG2*, *EGF*, *ERBB2*) with breast cancer risk and survival: the Breast Cancer Health Disparities Study. Breast Cancer Res Treat.

[25] Vaughn NH, Stepanyan H, Gallo RA, Dhawan A (2017). Genetic Factors in Tendon Injury: A Systematic Review of the Literature. Orthop J Sports Med.

[26] Kang X, Tian B, Zhang L, Ge Z, Zhao Y, Zhang Y. Relationship of common variants in *MPP7*, *TIMP2* and *CASP8* genes with the risk of chronic achilles tendinopathy. Sci Rep. 2019;9:17627. 10.1038/s41598-019-54097-y. 10.1038/s41598-019-54097-y PMC6879592 31772230

[27] Pruna R, Artells R, Ribas J, Montoro B, Cos F, Muñoz C, Rodas G, Maffulli N (2013). Single nucleotide polymorphisms associated with non-contact soft tissue injuries in elite professional soccer players: influence on degree of injury and recovery time. BMC Musculoskelet Disord.

