# Supplementary material for: Why PRP works only on certain patients with tennis elbow? Is PDGFB gene a key for PRP therapy effectiveness? A prospective cohort study
Source: BMC Musculoskelet Disord. 2021 Aug 18;22:710. doi: 10.1186/s12891-021-04593-y (PMC8375168; doi:10.1186/s12891-021-04593-y)
Supplement: Supplementary file 2 — Additional file 2: Platelets parameters and pain scores values in CC homozygotes and T allele carriers of the rs2285097 PDGFB gene polymorphism. [file 12891_2021_4593_MOESM2_ESM.docx]

**Additional file 2** Platelets parameters and pain scores values in CC homozygotes and T allele carriers of the rs2285097 *PDGFB* gene polymorphism.

| **Parameter** |  | **CC rs2285097** | | **CT+TT rs2285097** | | **p Mann-Whitney U test** |
| --- | --- | --- | --- | --- | --- | --- |
|  | week | median | ±QD | median | ±QD |  |
| Platelets parameters |  |  |  |  |  |  |
| PLT, 10^9^/l (WB) | 0 | 264.00 | 39.00 | 230.00 | 45.50 | 0.018 |
| PLT, 10^9^/l (PRP) | 0 | 364.00 | 77.50 | 341.00 | 66.00 | 0.329 |
| PDGF AB, ng/ml (PRP) | 0 | 8.77 | 3.33 | 8.06 | 2.21 | 0.299 |
| PDGF BB, ng/ml (PRP) | 0 | 4.20 | 0.61 | 5.09 | 1.57 | 0.275 |
| PROMs |  |  |  |  |  |  |
| VAS | 0 | 4.50 | 2.25 | 6.00 | 2.00 | 0.108 |
|  | 2 | 2.00 | 1.00 | 4.00 | 1.50 | 0.006 |
|  | 4 | 2.00 | 1.00 | 3.50 | 1.50 | 0.023 |
|  | 8 | 1.00 | 1.00 | 3.00 | 2.00 | 0.015 |
|  | 12 | 1.00 | 1.50 | 3.00 | 1.50 | 0.040 |
|  | 24 | 1.00 | 1.00 | 2.00 | 2.00 | 0.055 |
|  | 52 | 0.00 | 1.50 | 2.00 | 2.50 | 0.065 |
| ΔVAS (vs week 0) | 2 | 2.00 | 2.00 | 1.00 | 1.50 | 0.451 |
|  | 4 | 2.00 | 1.50 | 2.00 | 2.00 | 0.494 |
|  | 8 | 3.00 | 2.50 | 2.00 | 2.00 | 0.715 |
|  | 12 | 2.00 | 1.50 | 3.00 | 2.00 | 0.836 |
|  | 24 | 3.00 | 2.00 | 3.00 | 2.00 | 0.892 |
|  | 52 | 2.50 | 1.75 | 4.00 | 2.50 | 0.766 |
| QDASH | 0 | 52.27 | 8.57 | 52.27 | 13.64 | 0.411 |
|  | 2 | 27.27 | 11.36 | 43.18 | 17.05 | 0.025 |
|  | 4 | 25.00 | 10.23 | 37.50 | 14.77 | 0.031 |
|  | 8 | 13.64 | 12.50 | 34.09 | 17.05 | 0.025 |
|  | 12 | 13.64 | 10.23 | 29.55 | 18.18 | 0.024 |
|  | 24 | 6.82 | 11.36 | 29.55 | 20.45 | 0.039 |
|  | 52 | 11.36 | 17.05 | 22.45 | 22.73 | 0.087 |
| ΔQDASH (vs week 0) | 2 | 22.72 | 11.37 | 4.54 | 12.50 | 0.003 |
|  | 4 | 29.54 | 13.64 | 10.22 | 13.64 | 0.011 |
|  | 8 | 34.09 | 18.19 | 13.63 | 18.31 | 0.015 |
|  | 12 | 34.09 | 20.09 | 18.18 | 17.04 | 0.032 |
|  | 24 | 38.63 | 19.45 | 18.17 | 18.18 | 0.028 |
|  | 52 | 34.09 | 15.41 | 20.45 | 20.45 | 0.054 |
| PRTEE | 0 | 43.75 | 13.75 | 53.50 | 14.00 | 0.072 |
|  | 2 | 18.00 | 11.50 | 32.75 | 16.75 | 0.011 |
|  | 4 | 14.00 | 6.25 | 27.00 | 14.25 | 0.025 |
|  | 8 | 12.00 | 14.00 | 24.50 | 16.00 | 0.064 |
|  | 12 | 7.50 | 6.00 | 21.50 | 14.25 | 0.017 |
|  | 24 | 6.00 | 6.50 | 17.00 | 18.00 | 0.033 |
|  | 52 | 3.25 | 9.75 | 14.00 | 15.50 | 0.071 |
| ΔPRTEE (vs week 0) | 2 | 23.50 | 10.00 | 14.00 | 13.00 | 0.160 |
|  | 4 | 25.00 | 14.75 | 21.50 | 13.75 | 0.843 |
|  | 8 | 33.50 | 18.25 | 25.50 | 15.75 | 0.924 |
|  | 12 | 29.50 | 19.75 | 29.00 | 16.00 | 0.878 |
|  | 24 | 28.00 | 19.75 | 31.00 | 19.13 | 0.944 |
|  | 52 | 30.75 | 12.50 | 33.50 | 18.25 | 0.694 |

Legend: *PDGFB*, platelet-derived growth factor beta gene; QD, Quartile Deviation; WB, Whole Blood; PRP, Platelet-Rich Plasma; PROMs, patient-reported outcome measures; VAS, Visual Analog Scale; QDASH, quick version of Disabilities of the Arm, Shoulder and Hand score; PRTEE, Patient-Rated Tennis Elbow Evaluation.
