# Supplementary material for: Why PRP works only on certain patients with tennis elbow? Is PDGFB gene a key for PRP therapy effectiveness? A prospective cohort study
Source: BMC Musculoskelet Disord. 2021 Aug 18;22:710. doi: 10.1186/s12891-021-04593-y (PMC8375168; doi:10.1186/s12891-021-04593-y)
Supplement: Supplementary file 3 — Additional file 3: Platelets parameters, pain scores values and other characteristics differentiating AA homozygotes and G allele carriers of rs2247128 PDGFB gene polymorphism. [file 12891_2021_4593_MOESM3_ESM.docx]

**Additional file 3** Platelets parameters, pain scores values and other characteristics differentiating AA homozygotes and G allele carriers of rs2247128 *PDGFB* gene polymorphism.

| **Parameter** |  | **AA rs2247128** | | **AG+GG rs2247128** | | **p Mann-Whitney U test** |
| --- | --- | --- | --- | --- | --- | --- |
|  | week | median | ±QD | median | ±QD |  |
| Platelets parameters |  |  |  |  |  |  |
| PLT, 10^9^/l (WB) | 0 | 257.50 | 34.25 | 230.00 | 37.00 | 0.131 |
| PLT, 10^9^/l (PRP) | 0 | 333.00 | 110.50 | 349.00 | 66.00 | 0.618 |
| PDGF AB, ng/ml (PRP) | 0 | 8.66 | 3.32 | 8.18 | 2.27 | 0.953 |
| PDGF BB, ng/ml (PRP) | 0 | 4.72 | 0.72 | 4.64 | 1.53 | 0.865 |
| PROMs |  |  |  |  |  |  |
| VAS | 0 | 6.00 | 1.50 | 6.00 | 1.75 | 0.911 |
|  | 2 | 3.00 | 1.50 | 4.00 | 1.50 | 0.094 |
|  | 4 | 2.00 | 1.00 | 3.00 | 1.50 | 0.044 |
|  | 8 | 1.00 | 0.50 | 3.00 | 2.00 | 0.016 |
|  | 12 | 1.00 | 1.50 | 3.00 | 2.00 | 0.027 |
|  | 24 | 1.00 | 0.00 | 2.00 | 2.00 | 0.026 |
|  | 52 | 0.50 | 0.50 | 2.00 | 2.50 | 0.046 |
| ΔVAS (vs week 0) | 2 | 3.00 | 2.00 | 1.00 | 1.50 | 0.122 |
|  | 4 | 4.00 | 1.00 | 2.00 | 2.00 | 0.016 |
|  | 8 | 4.00 | 1.00 | 2.00 | 2.00 | 0.032 |
|  | 12 | 4.50 | 1.50 | 2.00 | 2.00 | 0.029 |
|  | 24 | 4.50 | 1.50 | 2.00 | 1.75 | 0.033 |
|  | 52 | 4.50 | 2.50 | 3.00 | 2.00 | 0.113 |
| QDASH | 0 | 53.41 | 9.09 | 52.27 | 13.64 | 0.631 |
|  | 2 | 36.36 | 18.52 | 40.91 | 15.91 | 0.591 |
|  | 4 | 32.95 | 15.91 | 36.36 | 14.77 | 0.276 |
|  | 8 | 18.18 | 15.91 | 34.09 | 17.61 | 0.042 |
|  | 12 | 13.64 | 11.36 | 29.55 | 19.89 | 0.048 |
|  | 24 | 10.23 | 9.09 | 28.41 | 21.59 | 0.120 |
|  | 52 | 6.82 | 6.82 | 20.45 | 23.86 | 0.109 |
| ΔQDASH (vs week 0) | 2 | 15.91 | 13.64 | 6.81 | 12.64 | 0.254 |
|  | 4 | 20.45 | 7.95 | 11.35 | 15.91 | 0.106 |
|  | 8 | 30.68 | 11.36 | 13.63 | 19.32 | 0.031 |
|  | 12 | 37.49 | 10.45 | 18.18 | 17.05 | 0.016 |
|  | 24 | 40.91 | 15.91 | 19.31 | 18.75 | 0.037 |
|  | 52 | 37.04 | 10.23 | 20.45 | 20.45 | 0.105 |
| PRTEE | 0 | 43.00 | 14.50 | 53.00 | 14.00 | 0.250 |
|  | 2 | 19.00 | 10.50 | 31.50 | 17.00 | 0.118 |
|  | 4 | 14.50 | 11.00 | 25.50 | 14.50 | 0.050 |
|  | 8 | 11.00 | 8.00 | 24.25 | 16.13 | 0.019 |
|  | 12 | 8.00 | 6.00 | 21.50 | 15.63 | 0.027 |
|  | 24 | 6.75 | 5.50 | 16.50 | 18.25 | 0.066 |
|  | 52 | 2.50 | 2.25 | 14.00 | 15.75 | 0.059 |
| ΔPRTEE (vs week 0) | 2 | 22.50 | 9.25 | 14.50 | 13.00 | 0.437 |
|  | 4 | 24.50 | 12.50 | 21.50 | 14.25 | 0.589 |
|  | 8 | 32.25 | 15.50 | 25.25 | 16.75 | 0.331 |
|  | 12 | 34.25 | 15.00 | 27.50 | 15.75 | 0.315 |
|  | 24 | 36.00 | 15.75 | 28.50 | 19.00 | 0.301 |
|  | 52 | 34.75 | 14.75 | 33.00 | 18.00 | 0.631 |

Legend: *PDGFB*, platelet-derived growth factor beta gene; QD, Quartile Deviation; WB, Whole Blood; PRP, Platelet-Rich Plasma; PROMs, patient-reported outcome measures; VAS, Visual Analog Scale; QDASH, quick version of Disabilities of the Arm, Shoulder and Hand score; PRTEE, Patient-Rated Tennis Elbow Evaluation.
