# Supplementary material for: Why PRP works only on certain patients with tennis elbow? Is PDGFB gene a key for PRP therapy effectiveness? A prospective cohort study
Source: BMC Musculoskelet Disord. 2021 Aug 18;22:710. doi: 10.1186/s12891-021-04593-y (PMC8375168; doi:10.1186/s12891-021-04593-y)
Supplement: Supplementary file 4 — Additional file 4: Platelets parameters and pain scores in CC homozygotes and G allele carriers of the rs5757572 PDGFB gene polymorphism. [file 12891_2021_4593_MOESM4_ESM.docx]

**Additional file 4** Platelets parameters and pain scores in CC homozygotes and G allele carriers of the rs5757572 *PDGFB* gene polymorphism.

| **Parameter** |  | **CC rs5757572** | | **CG+GG rs5757572** | | **p Mann-Whitney U test** |
| --- | --- | --- | --- | --- | --- | --- |
|  | week | median | ±QD | median | ±QD |  |
| Platelets parameters |  |  |  |  |  |  |
| PLT, 10^9^/l (WB) | 0 | 249.50 | 37.00 | 238.00 | 46.50 | 0.182 |
| PLT, 10^9^/l (PRP) | 0 | 338.00 | 110.50 | 349.00 | 66.00 | 0.975 |
| PDGF AB, ng/ml (PRP) | 0 | 9.17 | 2.82 | 8.06 | 2.21 | 0.457 |
| PDGF BB, ng/ml (PRP) | 0 | 4.82 | 0.62 | 4.45 | 1.58 | 0.820 |
| PROMs |  |  |  |  |  |  |
| VAS | 0 | 6.00 | 1.50 | 6.00 | 1.75 | 0.940 |
|  | 2 | 3.00 | 1.00 | 4.00 | 1.50 | 0.135 |
|  | 4 | 3.00 | 1.00 | 3.00 | 1.50 | 0.115 |
|  | 8 | 2.00 | 1.00 | 3.00 | 2.00 | 0.198 |
|  | 12 | 1.00 | 1.00 | 3.00 | 1.50 | 0.161 |
|  | 24 | 1.00 | 0.00 | 2.00 | 2.00 | 0.047 |
|  | 52 | 1.00 | 0.50 | 2.00 | 2.50 | 0.057 |
| ΔVAS (vs week 0) | 2 | 3.00 | 2.00 | 1.00 | 1.50 | 0.195 |
|  | 4 | 4.00 | 1.00 | 2.00 | 2.00 | 0.051 |
|  | 8 | 4.00 | 2.00 | 2.00 | 2.00 | 0.246 |
|  | 12 | 4.00 | 2.00 | 3.00 | 2.00 | 0.352 |
|  | 24 | 4.00 | 1.50 | 2.00 | 1.50 | 0.038 |
|  | 52 | 4.00 | 2.50 | 3.00 | 2.25 | 0.143 |
| QDASH | 0 | 54.54 | 9.09 | 52.27 | 13.64 | 0.596 |
|  | 2 | 38.64 | 13.64 | 40.91 | 15.91 | 0.809 |
|  | 4 | 34.09 | 14.77 | 36.36 | 13.64 | 0.548 |
|  | 8 | 27.27 | 18.18 | 34.09 | 18.18 | 0.298 |
|  | 12 | 18.18 | 10.23 | 29.55 | 18.18 | 0.153 |
|  | 24 | 9.09 | 9.09 | 29.55 | 20.45 | 0.080 |
|  | 52 | 9.09 | 6.82 | 21.59 | 23.30 | 0.088 |
| ΔQDASH (vs week 0) | 2 | 13.63 | 13.64 | 6.81 | 12.57 | 0.364 |
|  | 4 | 20.45 | 12.50 | 11.36 | 15.91 | 0.334 |
|  | 8 | 29.54 | 19.32 | 13.63 | 17.16 | 0.243 |
|  | 12 | 36.36 | 21.59 | 18.18 | 15.91 | 0.100 |
|  | 24 | 43.18 | 17.05 | 18.18 | 18.18 | 0.020 |
|  | 52 | 34.09 | 15.91 | 19.31 | 19.89 | 0.066 |
| PRTEE | 0 | 46.00 | 15.25 | 52.75 | 13.00 | 0.630 |
|  | 2 | 23.50 | 11.25 | 30.25 | 17.00 | 0.240 |
|  | 4 | 17.00 | 8.75 | 25.50 | 14.25 | 0.218 |
|  | 8 | 13.50 | 10.75 | 24.00 | 15.75 | 0.132 |
|  | 12 | 8.50 | 6.00 | 21.50 | 14.75 | 0.095 |
|  | 24 | 6.00 | 5.50 | 17.00 | 18.00 | 0.047 |
|  | 52 | 3.50 | 3.00 | 14.50 | 15.63 | 0.049 |
| ΔPRTEE (vs week 0) | 2 | 23.50 | 6.00 | 14.00 | 13.38 | 0.222 |
|  | 4 | 24.00 | 12.50 | 21.50 | 13.75 | 0.753 |
|  | 8 | 33.50 | 17.00 | 25.00 | 15.75 | 0.419 |
|  | 12 | 30.50 | 19.50 | 27.00 | 15.00 | 0.431 |
|  | 24 | 38.00 | 22.50 | 28.50 | 17.75 | 0.092 |
|  | 52 | 39.00 | 15.50 | 32.25 | 17.63 | 0.218 |

Legend: *PDGFB*, platelet-derived growth factor beta gene; QD, Quartile Deviation; WB, Whole Blood; PRP, Platelet-Rich Plasma; PROMs, patient-reported outcome measures; VAS, Visual Analog Scale; QDASH, quick version of Disabilities of the Arm, Shoulder and Hand score; PRTEE, Patient-Rated Tennis Elbow Evaluation.
