# Supplementary material for: Why PRP works only on certain patients with tennis elbow? Is PDGFB gene a key for PRP therapy effectiveness? A prospective cohort study
Source: BMC Musculoskelet Disord. 2021 Aug 18;22:710. doi: 10.1186/s12891-021-04593-y (PMC8375168; doi:10.1186/s12891-021-04593-y)
Supplement: Supplementary file 5 — Additional file 5: Platelets parameters and pain scores values in GG homozygotes and T allele carriers of the rs1800817 PDGFB gene polymorphism. [file 12891_2021_4593_MOESM5_ESM.docx]

**Additional file 5** Platelets parameters and pain scores values in GG homozygotes and T allele carriers of the rs1800817 *PDGFB* gene polymorphism.

| **Parameter** |  | **GG rs1800817** | | **GT+TT rs1800817** | | **p Mann-Whitney U test** |
| --- | --- | --- | --- | --- | --- | --- |
|  | week | median | ±QD | median | ±QD |  |
| Platelets parameters |  |  |  |  |  |  |
| PLT, 10^9^/l (WB) | 0 | 246.00 | 18.50 | 238.00 | 41.25 | 0.637 |
| PLT, 10^9^/l (PRP) | 0 | 300.00 | 91.75 | 345.00 | 68.00 | 0.507 |
| PDGF AB, ng/ml (PRP) | 0 | 8.66 | 5.52 | 8.06 | 2.40 | 0.758 |
| PDGF BB, ng/ml (PRP) | 0 | 5.55 | 0.79 | 4.32 | 1.51 | 0.327 |
| PROMs |  |  |  |  |  |  |
| VAS | 0 | 6.00 | 1.50 | 6.00 | 2.00 | 0.989 |
|  | 2 | 3.00 | 1.50 | 4.00 | 1.50 | 0.234 |
|  | 4 | 2.50 | 1.50 | 3.00 | 1.50 | 0.206 |
|  | 8 | 2.00 | 1.00 | 3.00 | 2.00 | 0.336 |
|  | 12 | 1.00 | 2.50 | 3.00 | 1.50 | 0.383 |
|  | 24 | 1.00 | 0.50 | 2.00 | 2.00 | 0.017 |
|  | 52 | 0.50 | 1.50 | 2.00 | 2.00 | 0.155 |
| ΔVAS (vs week 0) | 2 | 3.00 | 0.50 | 1.00 | 1.50 | 0.181 |
|  | 4 | 3.50 | 1.00 | 2.00 | 2.00 | 0.105 |
|  | 8 | 3.00 | 1.50 | 2.00 | 2.00 | 0.571 |
|  | 12 | 3.50 | 1.50 | 3.00 | 2.00 | 0.808 |
|  | 24 | 4.00 | 1.50 | 2.00 | 2.00 | 0.036 |
|  | 52 | 4.00 | 1.50 | 3.00 | 2.00 | 0.270 |
| QDASH | 0 | 55.68 | 7.98 | 52.27 | 14.32 | 0.903 |
|  | 2 | 32.95 | 17.05 | 40.91 | 15.91 | 0.453 |
|  | 4 | 29.55 | 10.23 | 36.36 | 13.64 | 0.317 |
|  | 8 | 25.00 | 23.86 | 34.09 | 19.32 | 0.497 |
|  | 12 | 11.36 | 25.00 | 29.55 | 17.05 | 0.350 |
|  | 24 | 6.82 | 2.27 | 26.14 | 20.45 | 0.028 |
|  | 52 | 11.36 | 10.23 | 20.45 | 23.86 | 0.360 |
| ΔQDASH (vs week 0) | 2 | 13.63 | 13.64 | 6.81 | 13.64 | 0.329 |
|  | 4 | 20.45 | 12.50 | 11.36 | 15.91 | 0.464 |
|  | 8 | 21.36 | 17.05 | 15.90 | 18.30 | 0.718 |
|  | 12 | 31.59 | 25.00 | 18.18 | 15.91 | 0.564 |
|  | 24 | 44.31 | 16.14 | 19.31 | 18.18 | 0.033 |
|  | 52 | 34.09 | 14.77 | 20.45 | 19.32 | 0.345 |
| PRTEE | 0 | 41.75 | 15.25 | 53.00 | 14.00 | 0.334 |
|  | 2 | 23.00 | 13.50 | 30.00 | 17.00 | 0.356 |
|  | 4 | 16.00 | 8.75 | 25.50 | 14.00 | 0.340 |
|  | 8 | 16.00 | 14.25 | 24.00 | 15.25 | 0.335 |
|  | 12 | 9.00 | 19.25 | 21.25 | 14.50 | 0.369 |
|  | 24 | 3.75 | 4.00 | 15.75 | 18.00 | 0.021 |
|  | 52 | 3.75 | 6.50 | 12.50 | 15.75 | 0.144 |
| ΔPRTEE (vs week 0) | 2 | 22.25 | 5.25 | 14.50 | 13.75 | 0.467 |
|  | 4 | 20.00 | 8.50 | 21.50 | 14.00 | 0.856 |
|  | 8 | 32.00 | 13.00 | 25.50 | 16.75 | 0.968 |
|  | 12 | 22.75 | 17.50 | 29.00 | 16.00 | 0.702 |
|  | 24 | 37.75 | 23.25 | 30.50 | 18.75 | 0.149 |
|  | 52 | 38.75 | 15.50 | 32.50 | 17.50 | 0.549 |

Legend: *PDGFB*, platelet-derived growth factor beta gene; QD, Quartile Deviation; WB, Whole Blood; PRP, Platelet-Rich Plasma; PROMs, patient-reported outcome measures; VAS, Visual Analog Scale; QDASH, quick version of Disabilities of the Arm, Shoulder and Hand score; PRTEE, Patient-Rated Tennis Elbow Evaluation.
