# Supplementary material for: Why PRP works only on certain patients with tennis elbow? Is PDGFB gene a key for PRP therapy effectiveness? A prospective cohort study
Source: BMC Musculoskelet Disord. 2021 Aug 18;22:710. doi: 10.1186/s12891-021-04593-y (PMC8375168; doi:10.1186/s12891-021-04593-y)
Supplement: Supplementary file 6 — Additional file 6: Platelets parameters and pain scores values in AA homozygotes and T allele carriers of the rs7289325 PDGFB gene polymorphism. [file 12891_2021_4593_MOESM6_ESM.docx]

**Additional file 6** Platelets parameters and pain scores values in AA homozygotes and T allele carriers of the rs7289325 *PDGFB* gene polymorphism.

| **Parameter** |  | **AA rs7289325** | | **AT+TT rs7289325** | | **p Mann-Whitney U test** |
| --- | --- | --- | --- | --- | --- | --- |
|  | week | median | ±QD | median | ±QD |  |
| Platelets parameters |  |  |  |  |  |  |
| PLT, 10^9^/l (WB) | 0 | 246.00 | 46.50 | 238.00 | 34.50 | 0.524 |
| PLT, 10^9^/l (PRP) | 0 | 328.00 | 80.00 | 351.00 | 63.50 | 0.446 |
| PDGF AB, ng/ml (PRP) | 0 | 7.88 | 2.13 | 8.37 | 2.24 | 0.385 |
| PDGF BB, ng/ml (PRP) | 0 | 4.82 | 1.29 | 4.29 | 1.57 | 0.536 |
| PROMs |  |  |  |  |  |  |
| VAS | 0 | 6.00 | 1.75 | 6.00 | 1.75 | 0.738 |
|  | 2 | 4.00 | 1.50 | 4.00 | 1.50 | 0.486 |
|  | 4 | 3.00 | 1.50 | 3.00 | 1.50 | 0.337 |
|  | 8 | 3.00 | 1.50 | 3.00 | 2.00 | 0.441 |
|  | 12 | 2.00 | 1.50 | 3.00 | 2.00 | 0.350 |
|  | 24 | 1.00 | 2.00 | 2.00 | 2.25 | 0.590 |
|  | 52 | 1.50 | 2.00 | 2.00 | 2.50 | 0.644 |
| ΔVAS (vs week 0) | 2 | 2.00 | 1.50 | 1.00 | 1.50 | 0.422 |
|  | 4 | 3.00 | 1.50 | 2.00 | 2.00 | 0.248 |
|  | 8 | 3.00 | 1.50 | 2.00 | 2.00 | 0.318 |
|  | 12 | 4.00 | 1.50 | 2.00 | 2.00 | 0.052 |
|  | 24 | 3.00 | 1.75 | 2.50 | 2.00 | 0.362 |
|  | 52 | 4.00 | 2.50 | 3.00 | 2.50 | 0.474 |
| QDASH | 0 | 54.54 | 12.50 | 52.27 | 13.64 | 0.732 |
|  | 2 | 39.77 | 15.91 | 40.91 | 15.91 | 0.798 |
|  | 4 | 35.23 | 13.64 | 36.36 | 15.91 | 0.900 |
|  | 8 | 29.55 | 14.77 | 34.09 | 20.45 | 0.156 |
|  | 12 | 27.27 | 15.91 | 29.55 | 20.45 | 0.195 |
|  | 24 | 15.91 | 21.59 | 28.41 | 23.30 | 0.566 |
|  | 52 | 15.91 | 19.32 | 20.45 | 25.00 | 0.927 |
| ΔQDASH (vs week 0) | 2 | 6.81 | 12.50 | 6.81 | 13.64 | 0.743 |
|  | 4 | 16.59 | 15.91 | 11.35 | 15.77 | 0.735 |
|  | 8 | 24.54 | 18.18 | 11.36 | 19.32 | 0.144 |
|  | 12 | 27.27 | 19.32 | 15.90 | 17.05 | 0.039 |
|  | 24 | 30.68 | 21.59 | 19.31 | 18.75 | 0.255 |
|  | 52 | 31.82 | 19.32 | 20.45 | 20.45 | 0.441 |
| PRTEE | 0 | 44.00 | 14.00 | 53.75 | 14.00 | 0.290 |
|  | 2 | 27.25 | 15.00 | 30.50 | 16.75 | 0.800 |
|  | 4 | 23.25 | 11.75 | 25.50 | 15.50 | 0.842 |
|  | 8 | 19.50 | 12.75 | 26.00 | 16.50 | 0.176 |
|  | 12 | 19.00 | 12.50 | 20.00 | 18.00 | 0.311 |
|  | 24 | 11.50 | 15.75 | 17.50 | 18.50 | 0.537 |
|  | 52 | 11.75 | 9.75 | 11.50 | 16.25 | 0.919 |
| ΔPRTEE (vs week 0) | 2 | 16.25 | 9.25 | 14.50 | 13.75 | 0.790 |
|  | 4 | 21.75 | 11.75 | 21.50 | 15.50 | 0.501 |
|  | 8 | 30.50 | 17.00 | 25.50 | 16.75 | 0.738 |
|  | 12 | 30.00 | 17.50 | 27.00 | 16.00 | 0.467 |
|  | 24 | 30.75 | 18.25 | 31.00 | 17.25 | 0.867 |
|  | 52 | 29.00 | 17.75 | 33.50 | 17.50 | 0.877 |

Legend: *PDGFB*, platelet-derived growth factor beta gene; QD, Quartile Deviation; WB, Whole Blood; PRP, Platelet-Rich Plasma; PROMs, patient-reported outcome measures; VAS, Visual Analog Scale; QDASH, quick version of Disabilities of the Arm, Shoulder and Hand score; PRTEE, Patient-Rated Tennis Elbow Evaluation.
